# The Antimicrobial Properties of Nanotitania Extract and Its Role in Inhibiting the Growth of *Klebsiella pneumonia* and *Haemophilus influenza*

**DOI:** 10.3390/antibiotics10080961

**Published:** 2021-08-10

**Authors:** Ahmad Mukifza Harun, Nor Farid Mohd Noor, Awatief Zaid, Mohamad Ezany Yusoff, Ramizu Shaari, Nor Dalila Nor Affandi, Fatirah Fadil, Mohd Azizi Abdul Rahman, Mohammad Khursheed Alam

**Affiliations:** 1 Engineering Faculty, Universiti Malaysia Sabah, Jalan UMS, Kota Kinabalu 88400, Malaysia; 2 Health Campus, School of Dental Sciences, Universiti Sains Malaysia, Kubang Kerian 16150, Malaysia; awatiefzaid@gmail.com (A.Z.); ezany@usm.my (M.E.Y.); ramizu@usm.my (R.S.); 3 Textile Research Group, Faculty of Applied Sciences, Universiti Teknologi MARA, Shah Alam 40450, Malaysia; dalila@uitm.edu.my (N.D.N.A.); fatirahfadil@uitm.edu.my (F.F.); 4 Malaysia Japan International Institute of Technology, Universiti Teknologi Malaysia, Jalan Sultan Yahya Petra, Kuala Lumpur 54100, Malaysia; azizi.kl@utm.my; 5 College of Dentistry, Jouf University, Sakaka 72721, Saudi Arabia; dralam@gmail.com; 6 Department of Dental Research Cell, Saveetha Dental College and Hospitals, Saveetha Institute of Medical and Technical Sciences, Chennai 600077, India

**Keywords:** nanoparticles extract, antimicrobial properties, *Klebsiella pneumonia*, *Haemophilus influenza*

## Abstract

Titanium dioxide (TiO_2_) is an antimicrobial agent which is considered of potential value in inhibiting the growth of multiple bacteria. *Klebsiella pneumonia* and *Haemophilus influenza* are two of the most common respiratory infection pathogens, and are the most. *Klebsiella pneumonia* causes fatal meningitis, while *Haemophilus* *influenza* causes mortality even in younger patients. Both are associated with bacteremia and mortality. The purpose of this study was to test a new antibacterial material, namely nanotitania extract combined with 0.03% silver that was developed at Universiti Malaysia Sabah (UMS) and tested against *K. pneumonia* and *H. influenza*. The nanoparticles were synthesized through a modified hydrothermal process, combined with molten salt and proven to have excellent crystallinity, with the band-gap energy falling in the visible light spectrum. The nanoparticle extract was tested using a macro-dilutional method, which involved combining it with 0.03% silver solution during the process of nanoparticle synthesis and then introducing it to the bacteria. A positive control containing the bacteria minus the nanoparticles extract was also prepared. 25 mg/mL, 12.5 mg/mL, and 6.25 mg/mL concentrations of the samples were produced using the macro dilution method. After adding the bacteria to multiple concentrations of nanoparticle extract, the suspensions were incubated for 24 h at a temperature of 37 °C. The suspensions were then spread on Mueller-Hinton agar (*K. pneumonia*) and chocolate blood agar (*H. influenza*), where the growth of bacteria was observed after 24 h. Nanoparticle extract in combination with silver at 0.03% was proven to have potential as an antimicrobial agent as it was able to inhibit *H. influenza* at all concentrations. Furthermore, it was also shown to be capable of inhibiting *K. pneumonia* at concentrations of 25 mg/mL and 50 mg/mL. In conclusion, the nanoparticle extract, when tested using a macro-dilutional method, displayed antimicrobial properties which were proven effective against the growth of both *K. pneumonia* and *H. influenza*.

## 1. Introduction

Bacterial infections are a leading cause of morbidity and mortality in the majority of septicemia cases [1]. Resistant Gram-negative nosocomial infections exacerbate the problems and highlight the inadequacy of current antibiotic treatments. In addition, the majority of the Gram-negative bacteria expressed Lipooligosaccharides (LOSs) on their lipids wall, hence boosting their resistance capability [2].

Klebsiella pneumonia *(K. pneumonia)* is a rod-shaped, lactose fermenting, Gram-negative bacterium commonly found in the skin, mouth, and intestinal tract [3]. Infection can lead to pneumonia, wound infection, meningitis, urinary tract infection, and septicemia. *K. pneumoniae* infections are typically “nosocomial” infections [4], which means they can easily be contracted in a hospital or healthcare setting. Individuals, who are ill or suffering from chronic diseases, or with reduced immune systems are more likely to acquire a *Klebsiella* infection [5].

Haemophilus Influenza *(H. influenza)* is also a Gram-negative bacterium containing pleomorphic coccobacilli [6]. It is a commensal organism found in the mucosal pharynx, eye, and genital tract, as well as a human-only pathogen that causes infections around the head and neck such as sinusitis, otitis media, conjunctivitis, and meningitis [7]. Encapsulated *H. influenza* serotype b (Hib) strains are considered to be the most pathogenic type in Europe [6].

Few agents, other than antibiotics have been proven to have bactericidal activities [8]. One exception is the silver ion, which has been demonstrated to interact with ribosomal subunit proteins, enzymes, and proteins of the bacterial organisms, to produce antimicrobial effects [9]. However, there is a paucity of studies on *K. pneumonia* and *H. influenza* infections. Titanium dioxide (TiO_2_) is a component of a semiconductor nanoparticle, which has antibacterial properties [10]. TiO_2_ acts as a pro-oxidant by producing reactive oxygen species (ROS), hence combatting the pathogenic bacteria. Our study proves that the combination of TiO_2_ with 0.03% of silver inhibits the growth of *K. pneumonia* and *H. influenza* in the growth plate, suggesting a bactericidal effect.

Nano titanium dioxide (nanoparticles) has been successfully extracted via a modified hydrothermal process [11,12,13]. It is well-known that titanium dioxide has a low energy band gap which enables excited electrons to jump from the valance band to the conduction band and to also easily perform photocatalytic activities. The manipulation of these low band gap activities has been performed in previous studies of dye-sensitized solar cells [14,15]. Titanium dioxide (TiO_2_) is currently being used against bacterial invasion. A reduction in *Escherichia* coli and Staphylococcus aureus in previous results when tested with a modified hydrothermal nanoparticle extract shows their capacity to inhibit the growth of these bacteria [8,16]. Furthermore, previous experiments with nano-titanium dioxide also resulted in the suppression of the bacteria’s mutagenic capability [17] and toxicological properties [18]. In addition, another study found that a nanoparticle extract when modified hydrothermally and subjected to molten salt synthesis using argentum nitrate salt at 3% by weight, was able to inhibit the growth of *Candida albicans* [19].

In recent decades, nanoparticles have been increasingly used in a variety of settings: the chemical, pharmaceutical, cosmetic, and food industries, as well as in air pollution control, photocatalysis and sewage treatment, and the manufacture of plastics, coatings, self-cleaning ceramics, self-cleaning glass, and antibacterial materials. The extensive production and use of nanoparticles have increased the risk of human exposure resulting in unknown health complications [20]. Nanoparticles enter into the human body via skincare products, nano-food, or nano-drug delivery applications. Due to their minute size, industrially released nanoparticles can also be inhaled as airborne particles. Consequently, concern about the safety and health implications of exposure to TiO_2_ has increased [21].

**Figure 1:**
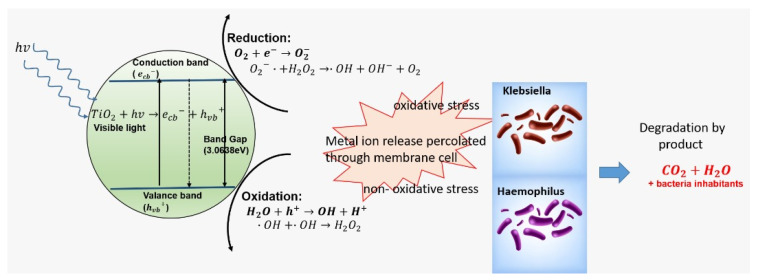
Photocatalytic mechanism.

The best way to describe the possibility of self-disinfection using nano-titanium dioxide is to describe it through a process called photocatalysis. Photocatalysis is a process that occurs when nano titanium dioxide is exposed to the photon energy source—which most research indicates ultraviolet light (UV). When exposure occurs, enough adsorption of the photons by nano titanium dioxide helps promote the excitation of electrons from the valance band ($e_{vb}-)$ to the conduction band ($e_{cb}-)$, leaving a positively charged hole called ($h_{vb}+)$ in the valance band. This is illustrated in Figure 1. This process of the photocatalysis equation can only happen when $hv\;\geq E_{band\;gap}$. The energy band gap plays an important role to promote the photocatalysis process. In most experiments and research papers, the most common nano titanium dioxide used is anatase with a band gap of 3.2 eV. This band gap falls in the ultraviolet spectrum, so it needs to be exposed to ultraviolet light in order for photocatalysis to occur. Our previous experiments [11,12,13], managed to produce a nano titanium dioxide with an energy band of 3.068 eV, which falls under the visible light spectrum range. Reactive oxygen species can be best explained via photodynamic therapy (PDT) and photothermal therapy (PTT), the two key strategies used in light-induced therapy [22,23]. In PDT, an interaction with a photosensitizer (PS), atmospheric oxygen, and photons is necessary. The photosensitizer absorbs light energy of a particular wavelength and becomes excited to the singlet state, before undergoing intersystem crossing to the triplet state. From the triplet state, it either transfers the energy to molecular oxygen (^3^O_2_) to form singlet oxygen (^1^O_2_) or interacts with biomolecules to generate other reactive oxygen species.

Unlike PDT, PTT does not require oxygen to target cells or tissues effectively: it has been used widely for antibacterial and anticancer treatment [15,16]. In PTT, light energy is converted into heat energy causing local heating that leads to cell death. The absorbed light causes excitation of PS energy, which is then rapidly released as vibrational energy in the form of heat. The heat generated, in turn kills the targeted cells, in what is clinically termed hyperthermia [23,24].

When photocatalysis occurs, electrons are free to migrate from the valence band to the conduction band leaving holes in the former and free-moving electrons in the latter. These holes can then be filled by migrating electrons from adjacent molecules, and the process of hole creation in the valance band is repeated. Furthermore, the flow of free electrons is observed in the conduction band, as a result of the migration from the valance band. Thus, both electrons and holes are mobile. The holes in the valance band can strongly oxidize with water molecules to generate reactive oxygen species (ROS), called OH^−^ and H^+^, resulting in the mineralization of pollutants and organic compounds. Concurrently, the reaction of electrons with oxygen forms a reactive oxygen species known as O_2_ at the surface of the conduction band.

These reactive oxygen species (ROS) can bring disorder to the bacteria/fungus or virus-cell metabolism and gene expressions. It is understood there are three possible ways of these reactive oxidation species (ROS) can destruct the cell membrane of bacteria, fungus, and viruses:

(i) oxidative stress induction; the reactive oxygen species (ROS), generated as a result of the redox process, favors the oxidation process in the cells that lead to the peroxidation of the lipid membrane and eventually attack proteins, depress the activity of certain periplasmic enzymes, and eventually interact with DNA and damage it [25].

(ii) metal ion release; where the metal ions released from metal oxide semiconductors are percolated through the cell membrane and directly interact with the –SH, -NH, and –COOH group of nucleic acids and proteins, which finally damages them. However, this method is less lethal than the other [26]. Since detailed studies of metal ion release on antimicrobial mechanisms have not been carried out yet, it is not considered as the major cause of cell death (Hussein-Al-Ali et al., 2014) [26,27].

(iii) the non-oxidative mechanism involves the inactivation of microbes by decreasing the critical cellular metabolism such as protein, amino acid, nucleotide, energy, and carbohydrate metabolism without oxidative stress induction [28]. After all the damages caused by these three possible membrane cell destructions, it degrades the products to simpler like salts, CO_2_, H_2_O with bacteria’s inhabitant.

## 2. Materials and Methods

The nanotitania extract was prepared together with a combination of multiple concentrations of silver at 0.01%, 0.03%, and 0.05%. As a negative control, an undoped sample of titanium dioxide was prepared without any added silver. One limitation was that the mixture could not be diluted in either water, dimethyl sulfoxide (DMSO), or sulphuric acid. Subsequent investigations were performed using a 0.03% silver nanotitania extraction (after considering its capability to prevent the growth of *K. pneumonia* and *H. Influenza*. The bacteria (*K. pneumonia* and *H. influenza*) were obtained from the stock culture at the Craniofacial Science Laboratory, School of Dental Sciences, Universiti Sains Malaysia. The nanoparticles extract was prepared at the Universiti Malaysia, Sabah, using TiO_2_ combined with silver at a concentration of 0.03%.

### 2.1. Nanoparticle Extraction Preparation

**Figure 2:**
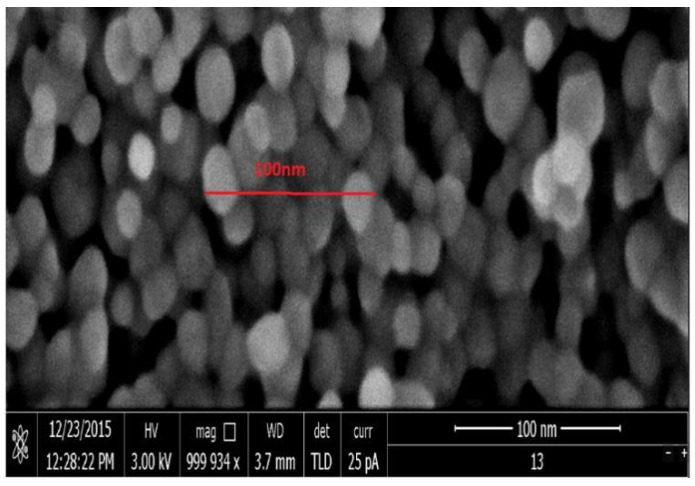
Growth morphology of nano TiO_2_.

The extraction of nanoparticles was performed by the previously described method [8]. The mix-ratio and the effect of molarity of the caustic hydrothermal activity produced optimal nanoparticle TiO_2_ growth conditions when embedded in ilmenite. Subsequently, argentum nitrate was mixed with TiO_2_ nanoparticles to produce highly crystalline nanoparticles in a molten salt synthesis, which provided dopant consolidation as in the samples. The materials were found to induce maximal growth at a certain temperature, acid–base concentration, and time as described in [8]. Besides the X-ray diffraction confirmation, the materials were also examined via Scanning Electron Microscopy (SEM) for size and shape conformity and Transmission Electron Microscopy (TEM) for nanoparticle crystallinity. Figure 2 shows the growth of TiO_2_ nanoparticles as observed under SEM with the circles, shape, and size within a 20–50 nm range.

**Figure 3:**
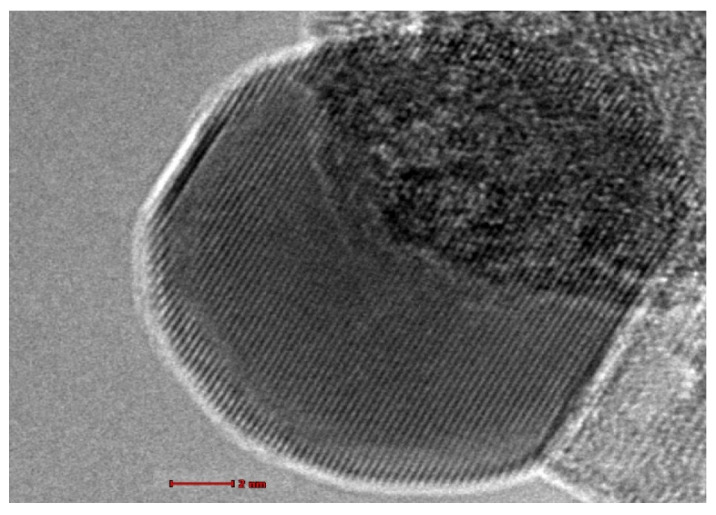
TEM image of nano TiO_2_.

Figure 3 shows the TEM results. The micrograph below clearly shows that the lattice fringes have extended to the edge of the nanoparticle and beyond to the surface of the nanocrystals. This micrograph shown is more informative than the TEM micrograph of pure TiO_2_ nanoparticles.

### 2.2. Antibacterial Activity Evaluation

A stock suspension of nanotitania at concentrations of 50 mg/mL was prepared by suspending 200 mg of 0.03% nanotitania powder in 4 mL of autoclaved distilled water. The mixture was subsequently stored in the incubator shaker for 24 h to ensure homogeneity. The stock suspension and four 15 mL conical tubes were exposed to ultraviolet (UV) light for 30 min in the safety cabinet for the purpose of sterilization. A sample of 2 mL of stock suspension was transferred to an empty conical tube using a pipette and labeled as 50 mg/mL (tube 1). Then, 1 mL of the 50 mg/mL solution from tube 1 was serially diluted into tubes 2 to 4 which contained 1 mL of distilled water. Finally, three samples of the concentration were produced at 25 mg/mL, 12.5 mg/mL, and 6.25 mg/mL.

Clinically isolated bacteria of *K. pneumonia* and *H. influenza* were received from the Microbiology and Parasitology Department, School of Medical Sciences, Universiti Sains Malaysia. The macrodilution method was used in this study for the determination of antibacterial properties of nanotitania suspension as described by CLSI, 2009, with some modifications [29]. It was considered more appropriate for determining the antibacterial properties of fastidious and non-fastidious bacteria than the diffusion method, which is not able to detect fastidious bacteria like *H. influenza* [30]. The bacteria colonies were suspended in 3 mL of Muller Hinton broth (MHB) and vortexed to create a smooth suspension. The turbidity of bacterial suspension was then adjusted to a 0.5 McFarland turbidity standard (1 × 10^8^ CFU/mL) using a densitometer. Then, 100 µL of bacterial suspension was diluted in 14.9 mL of MHB to obtain a final working concentration (1 × 10^6^ CFU/mL). One mL of bacteria suspension (1 × 10^6^ CFU/mL) was added into each well containing 1 mL of sample at different concentrations (50–6.25 mg/mL) to make a final concentration of 5 × 10^5^ CFU/mL. The broth containing only bacteria without TiO_2_ was used as a negative control. The suspensions were then incubated for 24 h, at 37 °C. A 50 µL of the mixture was transferred to the MH agar plate for *K. pneumonia* and chocolate blood agar plate for *H. influenza* and was spread using a hockey stick. After 24-h of incubation at 37 °C, the plates were observed for any sign of bacterial growth on the agar.

### 2.3. Toxicology Properties of TiO_2_

The mutagenic properties of the modified hydrothermal nanotitania extract were established using the Ames test for genotoxicity. The Ames test was performed on Salmonella strains (TA 98, TA 100, TA 1535, TA 1537, and TA 102) which contained mutations in several genes with and without S9 metabolic activation from rat liver using the standard assay. The materials were extracted in distilled water and serial dilutions of concentrations ranging from 313 to 5000 µg/mL were used after an incubation period of 24 h at 37 °C. These results suggested that none of the tested concentrations of the material extracts from the strains produced mutagenic effects, and findings from this study showed that the modified hydrothermal nanotitania extract was non-mutagenic under the described conditions [17].

## 3. Results

The results, as indicated in Table 1, demonstrate that nanotitania extract, when combined with 0.03% of silver, acts as an antimicrobial agent.

**Figure 4:**
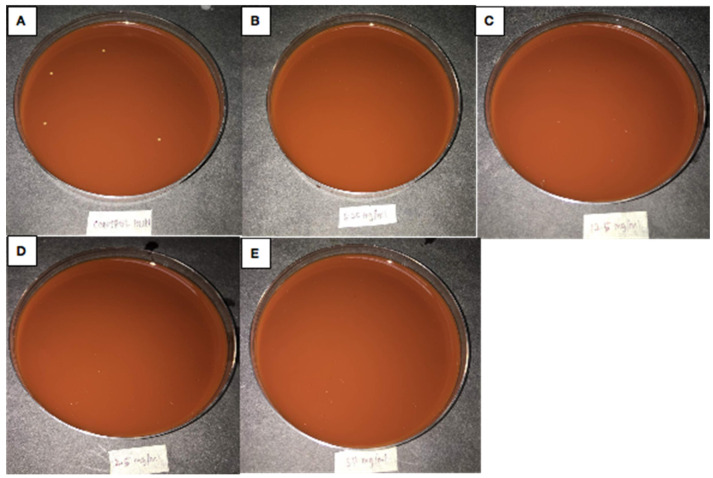
*H. influenza*. Negative control (**A**). 6.2 mg/mL TiO_2_ containing 0.03% silver (**B**). 12.5 mg/mL TiO_2_ containing 0.03% silver (**C**). 25 mg/mL TiO_2_ containing 0.03% silver (**D**). 50 mg/mL TiO_2_ containing 0.03% silver (**E**). It should be noted that there was no bacterial growth observed on the chocolate blood agar plates (**B**–**E**).

**Figure 5:**
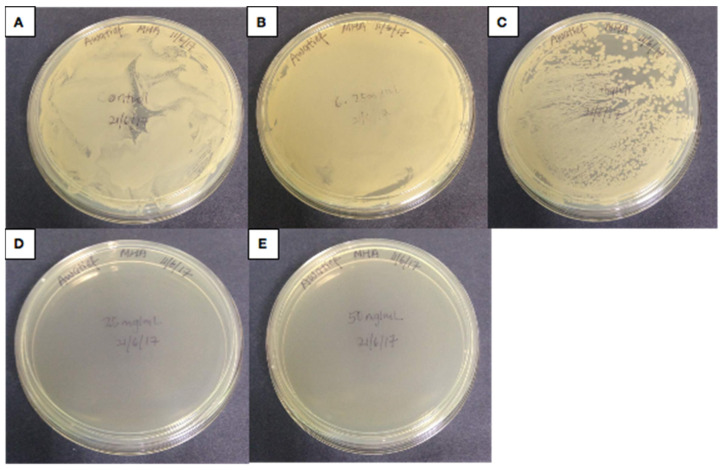
*K*. *pneumonia*. Negative control (**A**). 6.25 mg/mL TiO_2_ containing 0.03% silver (**B**). 12.5 mg/mL TiO_2_ containing 0.03% silver (**C**). 25 mg/mL TiO_2_ containing 0.03% silver (**D**). 50 mg/mL TiO_2_ containing 0.03% silver (**E**). It should be noted that there was no bacterial growth observed on the Mueller Hinton Agar plates (**D**,**E**).

The Nanoparticle and silver extract inhibited the growth of *H. influenza* on Chocolate blood agars at all concentrations; (50 mg/mL, 25 mg/mL, 12.5 mg/mL, and 6.25 mg/mL) as shown in Figure 4. The inability of *H. influenza* to grow on the plate at all concentrations (50−6.25 mg/mL), and on the Chocolate blood agar indicates that the Nanotitania silver has sufficient antibacterial properties to kill *H. influenza*. However, as Figure 5 illustrates, the *K. pneumonia* growth on the Mueller Hinton agar plates is only inhibited at concentrations of 25 mg/mL and 50 mg/mL. The 12.5 mg/mL and 6.25 mg/mL samples remained unchanged. These results indicate that Nanotitania silver only demonstrates antibacterial properties at 50 and 25 mg/mL concentrations (review-1-27).

## 4. Discussion

The antimicrobial potential of naturally sourced materials has encouraged extensive recent study in this area. This research was primarily designed to observe the growth inhibition of *K. pneumonia* and *H. influenza* on agar plates. Nanomaterials are effective against a broad spectrum of bacterial strains. According to several studies [31,32,33,34], metal oxides carry a positive charge, while microorganisms carry a negative charge, resulting in an electromagnetic attraction between them which leads to oxidization and the death of the microorganism, as shown in Figure 1. Silver nanoparticles have a tendency to aggregate because of their silver atom interaction. Prevention of this aggregation requires an inorganic carrier such as titanium oxide [35]. In this study, the focus was on the metal oxide, nanotitania (nanoTiO_2_), which has photocatalytic properties under visible light conditions. The use of 3% silver by mass was to ensure the crystallinity of the extracted nanoparticles, something that could not be guaranteed by employing the modified hydrothermal method alone.

However, the results in Figure 4 reveal the limits of *K. pneumonia* inhibition at concentrations of 25 mg/mL and 50 mg/mL. This result shows that the concentration of the solution is directly proportional to the inhibition reaction. This indicates that the higher the concentration of the nanotitania silver extract the more effective the inhibition mechanism reaction to the bacteria. This dissimilarity might also be due to the shape dependence of silver nanoparticles activity, suggesting further solution-phase method experiments [36]. The virulence capsule of *Klebsiella* is composed of complex acidic polysaccharides forming a thick bundle of fibrillous structures covering the bacterial surface in massive layers [37]. It protects the bacterium from polymorphonuclear granulocytes, phagocytosis, and prevents death through the release of bactericidal serum [38]. Apart from their antiphagocytic function, the *Klebsiella* polysaccharide capsule has been reported to inhibit the differentiation and functional capacity of macrophages in vitro [39]. The bacteria also produce multiple adhesins—fimbrial or nonfimbrial—which have distinct receptor specificity to augment the adherence of host cells, leading to a highly infectious state [40].

The results show that nanoparticles extract is an excellent antimicrobial agent against *K. pneumonia* and *H. influenza* infection. This study also supports the other theory as an excellent photocatalyst that acts as an antiseptic inhibiting bacterial reproduction and decomposing the cell membrane structure [41]. Furthermore, TiO_2_ nanoparticles are non-dissolved materials that have a long-lasting bactericidal effect, degrading organic contaminants [42]. A cytotoxicity study [43] showed that nanotitania with 0.05% silver in all concentrations did not show any adverse effects in a growth inhibition test. Hence, our nanotitania extract with 0.03% silver concentration has been proven to prevent cytotoxicity in humans.

In addition to these experiments, our sample has been sent to the SIRIM (Malaysia’s Productivity and Standard Board) for confirmation testing of the antimicrobial activities. The testing standard is listed below for the determination of the Minimum Inhibitory Concentration (MIC) and Minimum Bactericidal Concentration (MBC) by broth macrodilution.

*Preparation of Test Inoculum*

A loopful of microbial colonies from fresh plate culture was transferred into Tryptone water. The microbial inoculum size was adjusted using a spectrometer and further diluted to 10^6^ CFU/mL. A serial dilution was performed for microbial enumeration.

*Preparation of Sample in Test Medium*

4 mL of Mueller Hinton Broth (MHB) was transferred into test tubes 1 to 6. 4 mL of 100 mg/mL sample concentration was added into test tube 1 and the mixture was transferred to test tube 2 (this dilution step was repeated up to tube 4). 4 mL of the mixture was discarded from test tube 4, while test tube 5 was used as a positive growth control while test tube 6 was used as a sterile medium control. Another five sets were prepared.

*Inoculation of Test Microorganisms*

0.04 mL of prepared test inoculum was added to each of the test tubes except test tube 6. All test tubes were incubated at an optimal temperature as shown in Table 2 for 24 h, 48 h, and 72 h contact time.

*Enumeration of Inoculum Preparation*

1 mL mixture from test tube 5 was transferred into 9 mL of normal saline solution, and serial dilution was performed. Each dilution was plated and incubated using the medium and conditions specified in Table 2.

*Determination of MIC and MBC*

On completion of the contact time, each test tube of microorganisms was visually observed for microbial growth. The test tubes with clear broth indicated growth inhibition or no bacterial growth, and test tubes with turbid broth indicated growth promotion or bacterial growth. The MIC is determined as the lowest concentration of the sample where no microbial growth is observed. The MIC cannot be determined and is not valid when the sample itself is causing turbidity. 

After the contact time had elapsed, the MBC testwas performed by transferring 0.1 mL mixture from each tube and spreading it onto microbial medium and incubating it according to the conditions in Table 1. No growth observed on agar medium indicates zero cell viability, while agar medium with visible growth colonies indicates little or no reduction in cell viability.

As a suggestion for future work, researchers should focus on the study of nanotitania extract coatings on dental implants, surgical masks, and other medical equipment. A study by Kusy [43], proposed the modification of orthodontic wires made of stainless steel as bulk material and subsequently tested the photo-activated anti-adherent and antibacterial properties of their surfaces. Results showed that the bacterial mass bound to the TiO_2_-coated orthodontic wires remained unchanged after adhesion tests, whereas uncoated wires increased their mass by 4.97%. Furthermore, the bactericidal effect of TiO_2_-coated orthodontic wires inhibits the development of dental plaque, which is initiated by the adhesion of *S. mutans,* suggesting a specific device of interest [44].

## 5. Conclusions

Metal-containing nanomaterials are beneficial in dentistry as a means of infection control, but little is known about their antibacterial properties. In previous studies, TiO_2_ nanoparticles have been shown to have an inhibitory effect on the growth of *H. influenza* and *K. pneumonia,* and this was confirmed by our study. Hence, TiO_2_ nanoparticles can be considered potent antibacterial agents. Nanotechnology plays an important role in therapeutic procedures and diagnostics. It enables the precise and effective treatment of patients thanks to the considerable antibacterial properties of nanoparticles.

The reason for the use of 0.03% silver in this experiment was limited sample stock availability: the production of nano TiO_2_ is very time-consuming and can only be produced in a small quantities via synthesis. The Ag was doped with the nano TIO_2_ to produce the highly crystalline crystal without much alteration of the material properties, such as the composite composition. The benefit of using a hydrothermally modified nano TiO_2_ extraction is that the photocatalytic properties are activated under normal lighting conditions since the band gap falls within the visible spectrum.

## Figures and Tables

**Table 1:** The *H. influenza* and *K. pneumonia* growth were tested for 24 h.

Nanoparticles Extract Concentrations	*Haemophilus influenza*	*Klebsiella pneumonia*
150 mg/mL + 0.03% silver + bacteria	No growth	No growth
25 mg/mL + 0.03% silver + bacteria	No growth	No growth
12.5 mg/mL + 0.03% silver + bacteria	No growth	Growth
6.25 mg/mL + 0.03% silver + bacteria	No growth	Growth
Bacterial only (Negative Control)	Growth	Growth

**Table 2:** MIC and MBC assays of nanotitania against *Klebsiella pneumoniae* ATTC 4352.

Test Tube	Dilution (Ratio)	Sample Strength (mg/mL)	MIC	MBC		
				24 h	48 h	72 h
1	1:1	50	T	N	N	N
2	1:3	25	T	G	G	G
3	1:7	12.5	T	G	G	G
4	1:15	6.25	T	G	G	G
5	Positive control		T	G	G	G
6	Negative control		C	N	N	N
Test inoculum size			3.90 × 10^6^ CFU/mL			
Test tube 5: Purity check			Pure strain			
Test tube 5: Viable count			4.10 × 10^4^ CFU/mL			
Test tube 6: Medium sterility			Clear and sterile			

Notes: T (turbid broth test tube), C (clear broth test tube), N (no growth observed on agar medium), G (growth observed on agar medium), CFU (Colony forming unit).

## Data Availability

All data are available within the manuscript.
